# Comparison of Dominant and Non-Dominant Muscle Function Recovery and Limb Symmetry After Achilles Tendon Repair: A Retrospective Study

**DOI:** 10.3390/jcm15020707

**Published:** 2026-01-15

**Authors:** Xiangzi Xiao, Yonghwan Kim, Jiyoung Lee

**Affiliations:** 1 Department of Education, Taylor’s University, Jalan Taylor’s, Subang Jaya 47500, Malaysia; xiaoxiangzi@sd.taylors.edu.my; 2 Department of Physical Education, Gangneung-Wonju National University, Gangneung 25457, Republic of Korea

**Keywords:** strength, balance, limb symmetry index, range of motion, Achilles tendon

## Abstract

**Background/Objectives**: The Achilles tendon is the largest tendon in the human body; it is frequently ruptured during sports and other dynamic physical activities. The purpose of this study was to compare recovery patterns over time between injuries to the dominant (DMT) and non-dominant (NDMT) limbs, and to examine differences in the limb symmetry index (LSI) for the uninjured side. **Methods**: This study includes a retrospective analysis of individuals who completed a standard rehabilitation program for 12 months and had regular checkups every three months. The study sample comprised 17 patients with DMT injuries and 17 patients with NDMT injuries, all active male recreational participants who underwent surgical repair of an Achilles tendon rupture. Outcome measures included dorsiflexion range of motion (ROM), calf circumference, plantarflexion strength, hop test performance, and the Y-Balance Test (YBT). **Results**: Both groups demonstrated continuous, progressive improvement in ROM, plantarflexion strength, hop test distance, and YBT scores, showing a significant main effect of time (*p* < 0.05). Although DMT showed greater strength than NDMT at 6 and 9 months, this difference was no longer significant at 12 months (*p* > 0.05). In all three YBT directions, the DMT group achieved greater reach distances than the NDMT group at 12 months (*p* < 0.05). At the final follow-up, both groups exceeded 90% LSI in ROM, calf circumference, plantarflexion strength, and hop performance (*p* < 0.05). However, in the YBT, only the DMT group surpassed 90% LSI, whereas the NDMT group showed poorer recovery. **Conclusions**: ROM, calf circumference, and muscle strength ultimately showed no significant differences between groups, but dynamic balance recovery was superior in the DMT group. These findings suggest that clinicians and rehabilitation specialists should consider leg dominance when designing rehabilitation programs.

## 1. Introduction

The Achilles tendon, which connects the gastrocnemius and soleus muscles to the calcaneus, is the largest and strongest tendon in the human body. It plays a vital role in movement by transmitting the force needed for walking, running, and jumping [1]. Despite its strength, the Achilles tendon is one of the most frequently injured tendons in the lower limb. In recent years, the incidence of Achilles tendon ruptures has continued to rise. The rate increased from 20.7 cases per 100,000 people in 2009 to 26.5 cases in 2017, which is a trend largely attributed to the growing number of recreational participants engaging in high-intensity activities such as basketball, soccer, and tennis [2,3].

The most common and widely recommended treatment for an Achilles tendon rupture is early surgical repair. Surgical intervention is preferred, especially for individuals with high physical activity levels, because it lowers the risk of rerupture and allows an earlier return to movement compared with conservative treatment [4,5]. However, even after successful surgical repair, patients often experience long-term deficits in muscle strength, balance, and functional performance [6,7].

Among the functional deficits that persist after surgical repair, muscle weakness and strength asymmetry are the most prominent. In a study by Wenning et al., patients were followed for 3.5 years after surgery, and the researchers reported that the injured side continued to show more than a 10% reduction in muscle strength compared to the healthy side [6,8]. This can be attributed to the prolonged immobilization and reduced use of the limb following injury and surgery, which commonly lead to muscle atrophy and loss of strength. The gastrocnemius and soleus muscles, which generate plantarflexion torque, have been reported to show marked reductions in the cross-sectional area and contractile strength [9,10].

After an Achilles tendon rupture, postural stability and balance control are often impaired. The Achilles tendon not only serves as a mechanical linkage but is also a key component of proprioceptive feedback, contributing to the body’s ability to maintain balance during movement. Damage to the tendon and surrounding neuromuscular structures can disrupt sensory input, leading to deficits in joint position sense and reduced dynamic stability [11]. Bressel et al. reported a 27% loss of proprioception compared with healthy controls [12]. Tests such as the Star Excursion Balance Test and the Y-Balance Test (YBT) evaluate dynamic balance and limb symmetry. Patients who undergo Achilles tendon repair often exhibit greater postural sway and asymmetrical weight distribution [13,14]. Therefore, rehabilitation programs emphasize the benefits of progressive loading and strengthening exercises to restore full muscle function while also addressing neuromuscular weakness and proprioceptive deficits—highlighting the importance of balance-oriented training during recovery [15].

Previous researchers have explored various explanations for these asymmetries, including differences between the dominant and non-dominant limbs. Bohm et al. found that, although postoperative patients showed no significant differences between limbs in the tendon cross-sectional area or maximum strain, differences were identified in Young’s modulus and tendon length. Asymmetry indices ranged from 3% to 31% [16]. In a study by Gok et al., scores obtained from questionnaires revealed that the dominant leg showed better clinical outcomes than the non-dominant leg (*p* < 0.001). In total, 85.7% of participants returned to sports activities after an average of 5.82 months through early rehabilitation [17]. However, these findings are difficult to interpret definitively because postoperative recovery can be influenced by quality of rehabilitation, activity level, and individual behavioral factors, all of which can produce varied physiological and biomechanical outcomes [18,19]. As a result, the role of limb dominance in recovery after Achilles tendon repair remains unclear.

Therefore, this study aimed to evaluate the recovery of muscle function after Achilles tendon surgery, focusing specifically on whether the injured side is dominant or non-dominant. By comparing long-term postoperative outcomes between two groups, this study aims to determine whether the dominant limb influences the values and speed of functional recovery. The study hypothesis was that a difference exists in neuromuscular adaptation between the dominant and non-dominant sides after repair. This was based on previous research suggesting that structural differences may still exist even six years after repair [20]. These results are expected to provide insight into the neuromuscular adaptations that occur after surgery and help clinicians design more intensive and individualized rehabilitation programs.

## 2. Methods

### 2.1. Design and Participants

Participants were recruited from a specialized sports rehabilitation center that provides postoperative rehabilitation for lower-extremity injuries. This study was conducted retrospectively, targeting patients who visited the center and completed the examination over a 12-month period. Eligibility was confirmed through screening by an orthopedic surgeon and a certified sports rehabilitation specialist. Inclusion criteria were as follows: (1) male patients aged 20–45 years; (2) complete Achilles tendon rupture sustained during recreational sports activities such as basketball (5 participants), soccer (11 participants), badminton (8 participants), jogging (4 participants), and other sports (6 participants); (3) surgical repair completed within 2 weeks of injury; (4) initiation of standardized rehabilitation at the study facility within 4 weeks postoperation. Exclusion criteria included any history of Achilles tendon injury, neuromuscular or metabolic disease, or additional musculoskeletal conditions that could influence lower-limb function.

The sample size was calculated using G*Power 3.1.9.7 (Universität Düsseldorf, Düsseldorf, Germany) for a two-way repeated measures ANOVA. Based on the study conducted by Yan et al. [21], this study anticipated a medium effect size (f = 0.25) for the time × group interaction. With α = 0.05, power = 0.80, 4 repeated measurements, and an estimated correlation of 0.50 among repeated measures, the required sample size was determined to be 34 participants (17 per group) [22]. From the data pool, participants who completed rehabilitation and regular checkups over a 12-month period were divided into a dominant leg group (DMT, n = 17) and a non-dominant leg group (NDMT, n = 17), based on the most recent data. Limb dominance was identified from the preferred leg for kicking, in accordance with established lower-extremity dominance assessment protocols.

Postoperative management followed an integrated rehabilitation protocol including progressive weight-bearing, range-of-motion exercises, and stage-appropriate resistance training. Follow-up evaluations were conducted at 3, 6, 9, and 12 months after surgery. At each visit, participants completed a comprehensive range of assessments evaluating muscle strength, dynamic balance, and morphometric characteristics. Participants were instructed to avoid strenuous activity 24 h before testing. Data were collected and managed in accordance with institutional ethical guidelines. All participants provided written informed consent, and the study protocol received approval from the institutional review board of the affiliation.

### 2.2. Measurement

The assessments included dorsiflexion range of motion (ROM), isokinetic strength testing, the single-hop test, the Y-Balance Test (YBT), and calf circumference measurements. Participants completed a 5 min warm-up on a stationary bicycle, followed by light stretching of the calf and ankle muscles. For bilateral tests, the healthy limb was assessed first, followed by the surgically repaired limb. After measuring both sides, the limb symmetry index (LSI, %) was calculated using the following formula: injured side/uninjured side × 100. An LSI of 90% or greater (within ±10%) was considered within the normal range [23].

Tests related to the Achilles tendon involve the gastrocnemius and soleus muscles, with major parts including ROM, strength, and balance [18]. In this study, tests with high reliability and representativeness were selected. The ROM is dorsiflexion [24], and muscle strength was assessed using an isokinetic device, considered the gold standard [25], and a single hop test, which offers convenience and high functionality [26]. Finally, for balance assessment, there are two options: the 3-way YBT and the 8-way start test. In this study, the YBT was chosen due to its high reliability and ease of use [27].

#### 2.2.1. Active Dorsiflexion ROM and Calf Circumference

Active ankle dorsiflexion ROM was measured using a manual goniometer [28]. The model name of the device is a standard universal goniometer (Baseline^®^, Fabrication Enterprises Inc., Elmsford, NY, USA). The smallest markings on the protractor are in 1° increments, and every 5° is marked with a darker line. The universal goniometry for ankle dorsiflexion and plantarflexion is classified as having good to excellent test–retest reliability. Intraclass Correlation Coefficient (ICC) values ranged from 0.88 to 0.96, and 95% confidence intervals (CIs) were approximately 0.82–0.98 [24]. The patient was placed in the supine position to reduce tension in the gastrocnemius muscle. The ankle was placed in a neutral (0°) position. The goniometer was aligned with its axis centered over the lateral malleolus; the fixed arm was aligned with the fibular axis; and the movable arm was aligned with the fifth metatarsal. The participant gently and actively dorsiflexed the ankle without external resistance or discomfort. The angle between the two arms of the goniometer was recorded in degrees. Two measurements were taken, and the higher value was recorded.

**Figure 1:**
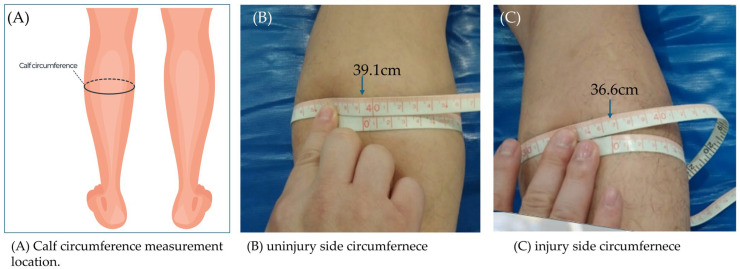
Calf circumference: (**A**) calf circumference measurement location; (**B**) uninjured side; (**C**) injured side.

Calf circumference was measured following Achilles tendon surgery to assess muscle atrophy and recovery [29] (Figure 1). The patient lay supine on a bed with the knees extended. The examiner used a flexible, non-stretchy tape measure to identify the point of maximum calf circumference, typically the thickest part of the gastrocnemius muscle. The tape measure was wrapped horizontally around the calf without compressing the skin, and constant tension was maintained. Measurements were recorded to the nearest 0.1 cm. The uninjured leg was also measured in the same manner to compare muscle symmetry and assess postoperative recovery.

#### 2.2.2. Strength: Isokinetic Strength Test and Single-Leg Hop Test

The equipment used included the CSMi dynamometer and HUMAC software (CSMi HUMAC NORM, Stoughton, MA, USA, version: HUMAC 2015). The reliability of the plantarflexion test has been studied, with ICC values, standard error of measurement (SEM), and minimal detectable change (MDC) ranging from 0.79 to 0.92, 5.2% to 13.0%, and 16.3% to 36.1%, respectively. The sampling frequency is 1000 Hz [30,31]. The dynamometer was calibrated according to the manufacturer’s instructions before each test session. The calibration procedure included system warm-up, mechanical zeroing of the lever arm, and verification of torque and angular position using the calibration routine built into the software. Accuracy was set for torque (±2.5 ft-lbs), position (±0.5 degrees), and time (0.0001 s) [32].

The isokinetic strength test for ankle plantarflexion and dorsiflexion is an objective method for assessing ankle joint musculature, particularly in patients. The procedure for this test was based on previous studies [33,34]. The participants were placed in the supine position on the dynamometer with their knee flexed at approximately 30°. The lower extremities, pelvis, and trunk were secured with straps to maximize the activity of the target muscles and minimize extraneous movement. In this analysis, only plantarflexion related to the Achilles tendon was analyzed.

The test foot was placed on the measurement adapter and securely fastened to the footplate with straps to prevent unintended movement. The ankle joint’s axis of rotation was aligned with the central axis of the machine by centering it over the lateral malleolus. Measurements were conducted within the manufacturer’s suggested test range: 10–20° of dorsiflexion and 40–50° of plantarflexion. Concentric contractions were performed four times at an angular velocity of 30°/s to measure peak torque. Participants completed a sufficient number of practice trials to familiarize themselves with the machine, followed by a one-minute rest period before the official test. Gravity correction was performed to offset the values caused by the weight of the limb and adapter. Participants remained passive except for the moment when maximum force was applied. The adapter was set to move from dorsiflexion to plantarflexion, and the weight at rest was measured at approximately 45°. Peak torque (Nm) was automatically calculated using the system software.

Single-hop test: To assess the function of the Achilles and calf muscles [35], the participant stands on the test leg at a starting line, performs a maximal forward hop, and lands stably on the same limb. The maximum distance, measured in centimeters from the starting line to the heel, is recorded after two successful attempts.

#### 2.2.3. Dynamic Balance

The YBT was administered to evaluate participants’ dynamic balance and postural control capabilities. This assessment was conducted using a dedicated Y-Balance Test kit (Move2Perform, Evansville, IN, USA) [27]. The ICCs for this test were as follows: anterior: 0.85–0.91 and 95% CI = 0.78–0.95; PM: 0.88–0.94 and 95% CI = 0.82–0.97; and PL: 0.86–0.93, 95% CI = 0.80–0.96 [36]. The standard error of measurement (SEM) and minimal detectable change (MDC) are as follows: anterior: 1.0–1.5 cm and 4.0–4.5 cm; PM: 1.5–2.0 cm and 6.0–6.5 cm; PL: 1.5–2.0 cm and 6.0–6.5 cm [37]. Participants wore comfortable athletic clothing and performed the test barefoot for natural movement of the feet and ankles. They received instruction on the proper testing technique and completed multiple practice trials to minimize any learning effect during the actual measurements. The YBT required subjects to maintain a single-leg stance on the testing leg while reaching with the opposite leg along three specified measurement lines oriented at 120° angles: anterior, posteromedial (PM), and posterolateral (PL). The goal was for the participant to reach as far as possible in each direction while maintaining their balance. They were instructed to lightly touch the reach indicator with the most distal part of their foot, avoiding any excessive weight shift or loss of balance. Reach distance was recorded to the nearest 0.5 cm. A trial was considered false and retaken if the participant shifted their weight onto the reaching leg, lost their balance, or failed to return to the starting position successfully. Three successful trials were performed in each of the three directions.

### 2.3. Surgical Procedures

The surgery was performed by a single orthopedic specialist using a mini-open repair technique, and is outlined below [5]. The patient was placed in the prone position, and the affected lower extremity was prepared and draped in a standard sterile fashion. A slight medial longitudinal skin incision was made over the Achilles tendon. Careful dissection was performed to expose and preserve the paratenon, which was incised longitudinally. The rupture site of the Achilles tendon was identified, and hematoma and degenerated tissue were debrided as necessary. The tendon ends were approximated, and tendon tension was adjusted to restore appropriate length and alignment. The tendon was repaired using a synovial suture technique, followed by meticulous paratenon closure to promote tendon healing and gliding. After confirming adequate repair stability, the wound was closed in layers. A short-leg splint was applied with the ankle positioned in slight plantarflexion. The procedure was then completed after checking for complications.

### 2.4. Protocol of Standard Rehabilitation

Postoperative rehabilitation followed a structured protocol that was designed to restore ankle mobility, calf muscle strength, and functional performance (Table 1) [19,38]. During Phase I (0–4 weeks), patients maintained the ankle in a plantarflexed position and progressed from non-weight-bearing to partial weight-bearing as tolerated. Gentle range-of-motion exercises were initiated within a safe range while avoiding excessive dorsiflexion to protect the repaired tendon. In Phase II (4–8 weeks), controlled ankle mobility, isometric calf activation, and gradual increases in weight-bearing were introduced. Between 8 and 12 weeks (Phase III), rehabilitation focused on restoring basic neuromuscular control through isotonic strengthening, gait training, proprioceptive exercises, and stationary cycling. Phase IV (3–6 months) emphasized improved tendon load capacity and limb symmetry with progressive resistance training, eccentric calf exercises, balance training, and functional movement tasks. Plyometric training and light jogging were incorporated only after patients demonstrated adequate strength and quality of movement. During Phase V (6–12 months), high-level plyometric exercises, agility drills, and sport-specific activities were implemented to restore explosive power and dynamic stability. Participants were instructed to exercise for at least 60 min, three or more days a week, and to contact the researchers via a hotline if they experienced any issues, including pain [39].

### 2.5. Data Analysis

Statistical analyses were performed using IBM SPSS Statistics version 22 (IBM Corp., Armonk, NY, USA). Descriptive statistics were used to summarize the general characteristics of the participants. Differences in categorical variables between groups were examined using chi-square tests. Independent *t*-tests were conducted to compare continuous variables between the two groups, and paired *t*-tests were used to evaluate within-group changes over time by comparing pre- and post-intervention outcomes. For dependent variables where the *t*-test results were statistically significant, the effect size was calculated using Cohen’s *d.* In addition, a two-way repeated-measures ANOVA was applied to assess interaction effects between time and group. The figures show the main effects of time (T) and group (G), as well as the interaction effect (T × G), F value, and degrees of freedom. Significant main or interaction effects were reported, and the level of statistical significance was set at *p* < 0.05 for all analyses.

## 3. Results

### 3.1. General Characteristics

Table 2 shows the general characteristics of the DMT and NDMT groups. There were no significant differences in age, height, or weight. The left side was the most common site of injury in the DMT group (5 vs. 12), while the right side was the most common in the NMDT group (*p* < 0.01). The visual analog scale (VAS), which measures current pain, showed that most patients in both groups reported mild pain. When asked about the circumstances surrounding the Achilles tendon rupture, 16 patients in the DMT group and 14 patients in the NMDT group reported non-contact injuries, without collision with others or objects (*p >* 0.05).

### 3.2. Comparison of ROM and Circumference Values with LSI

**Figure 2:**
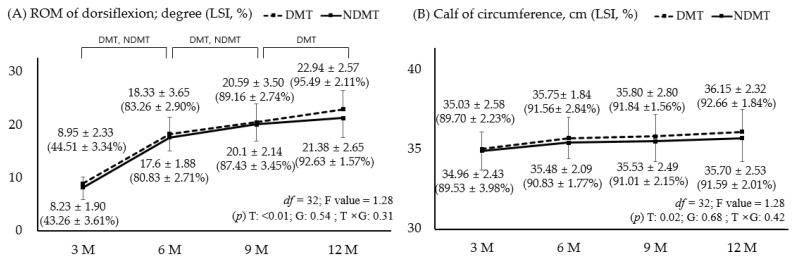
Range of motion for dorsiflexion (**A**) and calf circumference (**B**) in both groups; *p* < 0.05. DMT is represented by a dotted line and positive error bars; NDMT is represented by a straight line and negative error bars. DMT, dominant side; NDMT, non-dominant side; LSI, limb symmetry index; ROM, range of motion; T, time; G, group; M, months; df, degree of freedom.

Figure 2 shows the ROM and calf circumference between the participant groups. First, dorsiflexion, which indicates Achilles stiffness, showed continuous improvement in both groups up to 9 months. At the final 12-month follow-up, only the DMT showed improvement; however, this difference was not significant (*p* > 0.05). At 12 months, the LSI remained lower compared to the uninjured side with 95.49% for the DMT and 92.63% for the NDMT; however, this difference was not significant (*p* > 0.05). Calf circumference, which reflects muscle atrophy due to long-term disuse, decreased by approximately 2–3 cm from injury to postoperative recovery and showed a tendency to improve over time, with no significant difference between groups (*p* > 0.05). At 12 months, the LSI recovered to within 10% in both groups. While there were no significant differences at the 3-month follow-up, the main effect of time showed a significant change (*p* = 0.02).

### 3.3. Comparison of Plantarflexion Strength and Single-Hop Test Values with LSI

**Figure 3:**
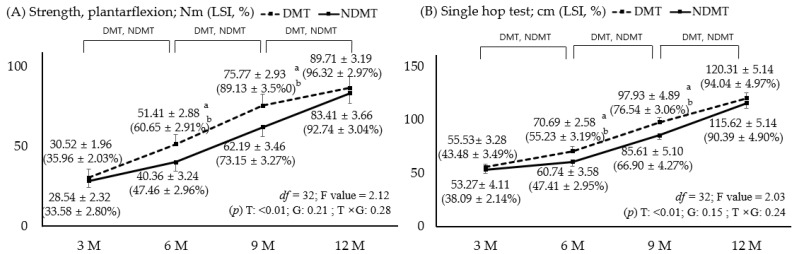
Plantarflexion strength (**A**) and single-hop test (**B**); *p* < 0.05. DMT is represented by a dotted line and positive error bars; NDMT is represented by a straight line and negative error bars. a and b indicate the significance of the independent *t*-test. The value is represented by a, and LSI is represented by b. DMT, dominant side; NDMT, non-dominant side; LSI, limb symmetry index; T, time; G, group; M, months; df, degree of freedom.

Figure 3 illustrates improvements in strength-related variables, including isokinetic plantarflexion strength and the single-hop test, which measures the jumping distance of a single leg. Both variables showed significant improvements up to 12 months with identical results (*p* < 0.05). At 6 and 9 months, DMT showed significantly higher values than NDMT, and LSI was also higher in DMT. There was a significant main effect of time; however, this was not significant between groups or for the interaction effect between time and group (*p* > 0.05). In both groups, plantarflexion strength and LSI ultimately reached over 90% of the normal range (±10%) in the single-hop test, indicating a return to normal strength. For plantarflexion, the effect size at 6 months was 3.83 (95% CI: 2.12–6.21) for strength value and 4.53 (95% CI: 2.44–6.87) for LSI; at 9 months, the strength value was 4.21 (95% CI: 3.12–5.98) and the LSI was 4.56 (95% CI: 3.40–6.02). In the hop test, the effect size at 6 months was 3.88 (95% CI: 2.10–6.40) for the hop test value and 6.97 (95% CI: 3.69–7.80) for the LSI; at 9 months, the hop test value was 2.46 (95% CI: 1.15–4.25) and the LSI was 2.59 (95% CI: 1.43–3.87).

### 3.4. Comparison of YBT Values with LSI

**Figure 4:**
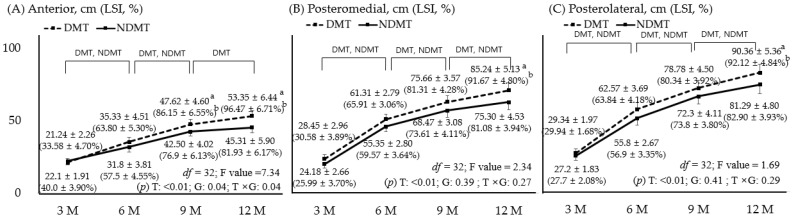
Y-Balance Test: anterior (**A**), posteromedial (**B**), posterolateral (**C**); *p* < 0.05. DMT is represented by a dotted line and positive error bars; NDMT is represented by a straight line and negative error bars. a and b indicate the significance post hoc. The value is represented by a, and LSI is represented by b. DMT, dominant side; NDMT, non-dominant side; LSI, limb symmetry index; T, time; G, group; M, months; df, degree of freedom.

Figure 4 compares dynamic balance ability across time and groups. Both groups improved in all three directions up to 9 months, though only the DMT showed improvement at 12 months (*p* < 0.05). There were significant differences between groups for the forward test at 9 and 12 months, as well as the PM and PL at 12 months. The 12-month follow-up revealed that the LSI of the PM and PL in the NDMT showed lower recovery compared to the unaffected side and the DMT (*p* < 0.05). In the anterior direction, the effect size at 9 months was 1.18 (95% CI: 0.58–2.97) for the anterior value and 1.45 (95% CI: 0.74–3.50) for LSI. At 12 months, the anterior value was 1.24 (95% CI: 0.66–2.84) and the LSI was 2.25 (95% CI: 1.15–4.01). In the PM, the effect size at 12 months was 2.05 (95% CI: 1.15–4.08) for the PM value and 2.41 (95% CI: 1.88–5.02) for LSI. In the PL, the effect size at 12 months was 1.78 (95% CI: 0.87–3.41) for the PL value and 2.09 (95% CI: 1.17–4.17) for LSI.

## 4. Discussion

This study investigated the differences in muscle function recovery and symmetry between DMT and NDMT after Achilles tendon surgery during a 12-month rehabilitation period. One key finding was that both groups showed significant improvements in dorsiflexion ROM and calf circumference over time, with no significant difference between DMT and NDMT. These results suggest that standardized rehabilitation of the repaired Achilles tendon should result in similar long-term outcomes for both the DMT and NDMT.

Recovery of ankle dorsiflexion ROM is an important indicator of functional recovery after tendon surgery, as it reflects flexibility and tendon elongation [18]. In this study, both groups showed a substantial increase in dorsiflexion ROM between 3 and 12 months, with the LSI exceeding 90% at 1 year. This aligns with previous studies showing that most patients undergoing structured rehabilitation achieve nearly symmetrical ankle ROM within 9 to 12 months post-surgery [40,41]. While some studies occasionally set the LSI criterion at >95%, considering the high skill level of the athletes [42,43], this study used a 90% criterion because the participants were recreational players.

While some clinicians recommend maintaining immobilization for up to 12 weeks to stabilize the surgical site, prevent tendon elongation, and avoid complications, other specialists state that initiating early mobilization movements and gentle stretching between 4 and 6 weeks post-surgery is not only safe but promotes better recovery by facilitating faster healing and preventing stiffness [18]. The debate regarding Achilles stiffness in DMT and NDMT remains ongoing. Salman et al. investigated differences in Achilles stiffness in healthy individuals, suggesting that DMT may exhibit higher levels of stiffness than NDMT due to greater use and loading [44].

Calf circumference represents the cross-sectional area of the muscle. It is used to measure muscle atrophy caused by prolonged disuse and serves as an indicator of recovery. In many cases, it decreases by approximately 2–3 cm during the recovery period from injury to post-surgery [45]. The results of this study showed that the circumference gradually improved, reaching over 90% symmetry at the 12-month mark. Furthermore, no difference existed between DMT and NDMT. A slight residual deficit persisted, but this remained within the range commonly reported after Achilles tendon surgery [46]. Even after 12 months, calf circumference did not fully recover. Similar findings have been reported in other studies; for instance, Rosso et al.’s study with an average follow-up of 91 months still found that a reduction in muscle mass and circumference persisted postoperatively [47].

Plantar flexion strength was assessed using isokinetic equipment for plantarflexion and the single-hop test. Ultimately, both DMT and NDMT achieved over 90% recovery of LSI compared to the unaffected side. However, at 6 and 9 months, DMT had a higher LSI than NDMT. Borges et al. suggested that long-term asymmetry in plantar function after Achilles surgery can persist for over a year [48]; as such, this study’s observation of continuous strength improvement over one year is consistent with many previous studies [49,50]. Despite involving a different surgery, a study focused on the continuous observation of patients with anterior cruciate ligament (ACL) rupture also found that at 6 months post-surgery, DMT patients had stronger quadriceps than NDMT patients. However, no differences were observed between groups at 12 months [51]. Another study on ACL tear patients also reported that the recovery process and level of muscle strength are unrelated to leg dominance, specifically noting no difference in the time patients took to return to play [52]. However, a long-term 2-year follow-up study of Achilles tendon rupture patients by Gok et al. found that DMT rupture patients demonstrated higher recovery capacity compared to NDMT [17].

Finally, this study investigated the recovery of lower limb balance using the YBT. This recovery pattern differed from that of muscle strength, showing gradual improvement up to 12 months. This is consistent with previous reports indicating steady neuromuscular recovery of the Achilles tendon–gastrocnemius–soleus complex over several months post-surgery [53]. Ultimately, DMT reached a greater distance and demonstrated a lower LSI than NDMT; the multi-directional reach test is influenced by comprehensive lower limb capabilities, including ankle stability, dorsiflexion ROM, hip condition, and proprioceptive sensation [54,55]. Therefore, the advantage of DMT lies in familiarity and comfort with leg use across diverse environments, and repeated engagement in these activities may indicate faster recovery of neuromuscular coordination and sensorimotor processing, which are commonly impaired after Achilles tendon rupture [56]. However, several review articles express concern about taking a definitive stance on balance differences between DMT and NDMT [57,58]. DMT is the difference in proprioception between the dominant and non-dominant sides. Studies conducted on healthy adults have shown that proprioception is stronger on the non-dominant side than on the dominant side, and this phenomenon is more pronounced in the lower limbs [59,60]. However, our study showed high results in the DMT at 9 and 12 months, which may be due to the effect of intensive training on the injured area after surgery. Furthermore, since the unaffected side was tested first during the examination, it is important not to overlook the possibility of learning effects and feed-forward motor control adaptations occurring on the affected side [61].

Notably, in this study, a significant difference was observed between groups in the anterior direction of DMT. This may have been influenced by differences in posture when performing tests. Moving in the anterior direction requires the body’s center of gravity to shift backward, necessitating the heel of the standing foot to remain on the ground while demanding significant dorsiflexion. Although not statistically significant at 12 months, the dorsiflexion ROM in this study was slightly lower in the NDMT; however, this may have been influenced by the anterior YBT. A mechanical analysis by Hoch et al. also reported a significant correlation between the anterior YBT and dorsiflexion ROM, supporting our findings [62].

In our study, the left side was most frequently injured in the NDMT group. However, previous studies have reported no significant difference in the rate of injury between the left and right feet [63]. The several participants sustained injuries during soccer and badminton. Due to the nature of these sports, the left foot is more likely to be injured, as it is the first foot to land after a jump in badminton, and it serves as the supporting foot for right-footed kicks in soccer [64,65].

These findings provide important clinical insights into muscle function recovery and limb symmetry after Achilles tendon surgery, particularly for the dominant limb. Both the DMT and NDMT limbs showed gradual improvement over 12 months, but significant, albeit small, differences emerged in muscle strength, functional performance, and dynamic balance. These differences may provide useful information for rehabilitation planning, decisions regarding return to sports, and long-term prevention of injury.

This study has several limitations that should be accounted for when interpreting the results. First, the sample consisted solely of male patients aged 20 to 45 who experienced Achilles tendon ruptures during recreational sports activities. Therefore, these findings cannot be generalized to women, older individuals, competitive athletes, or those with non-sports-related injuries. Limb dominance was determined solely based on self-reported leg preference, which, while widely used, may not fully capture functional dominance or task-specific asymmetry. The single-center nature of this study, conducted at one rehabilitation center, also introduces potential performer-related bias. Despite standardized protocols, subtle differences in rehabilitation progression, patient compliance, and training emphasis may have influenced recovery patterns. The statistical power calculated during the sample size determination process was approximately 0.5, which suggests a risk of committing a Type II error. Additional limitations include the fact that retrospective studies do not have the same level of scientific rigor as blinded outcome studies, the lack of a rehabilitation adherence assessment, and an imbalance of injury sides between groups. In particular, retrospective studies may be subject to an increased risk of researcher bias due to inherent limitations in the study design, and there is a possibility of errors in measurement tools and by the researchers themselves. Despite these limitations, this study provides meaningful evidence regarding the influence of limb dominance on recovery patterns after Achilles tendon surgery and offers valuable clinical insights for postoperative rehabilitation planning.

## 5. Conclusions

This retrospective study examined muscle function recovery and limb symmetry between DMT and NDMT after Achilles tendon surgery. Both groups demonstrated significantly improved ROM, calf circumference, plantarflexion strength, and functional performance over the 12-month period. DMT showed superior strength and jumping performance mid-rehabilitation, but differences were not evident by the final follow-up. Both groups achieved >90% LSI, and differences in dynamic balance at 12 months followed a distinct trend from muscle strength.

## Figures and Tables

**Table 1:** Standard rehabilitation and care after Achilles tendon repair.

Stage	Period	Contents	Goal
Phase I	0–4 week	Immobilization with a cast Tolerable toe movement and leg lift	heeling wound
Phase II	4–8 week	Soft brace and partial weight bearing Start range of motion and mobilization	achieve range of motion
Phase III	8–12 week	Gradual progression from partial weight-bearing to full weight-bearing Mild range of motion exercise	start weight bearing
Phase IV	3–6 month	Gradual plantarflexion strength, heel rises, and jumps Gradual regain of balance and functional exercise	restore strength
Phase V	6–12 month	Progress to full power and function Progress to stability Restore sports-related activity and skill	normal functioning

**Table 2:** General information of participants.

Variables	DMT (*n* = 17)	NDMT (*n* = 17)	*t* or χ^2^	*p*-Value
Age, years	32.43 ± 3.81	31.3 ± 4.14	1.48	0.53
Height, cm	176.64 ± 3.55	177.9 ± 3.93	1.19	0.81
Weight, kg	74.23 ± 5.56	72.8 ± 4.80	1.29	0.32
Player career, years	12.31 ± 3.27	13.7 ± 3.87	−1.88	0.30
Injury side, *n* (%)				
left/right	5/12	14/3	9.66	<0.01
VAS, *n* (%)				
0–3/4–6/>7	12/3/2	13/3/1	0.37	0.83
Injury situation, *n* (%)				
contact/non-contact	1/16	3/14	1.13	0.60

*p* < 0.05; Abbreviations: DMT, dominant; NDMT, non-dominant; VAS, visual analog scale.

## Data Availability

The data presented in this study are available upon reasonable request from the corresponding author.
